# Alterations in urinary metals following pulsed 660 nm photobiomodulation in a pediatric patient with autism spectrum disorder: a case report

**DOI:** 10.3389/fped.2026.1819736

**Published:** 2026-06-23

**Authors:** Alex Zaharakis, Christian Bogner

**Affiliations:** Autism is Biomedical Foundation, Farmington Hills, MI, United States

**Keywords:** aluminum, autism spectrum disorder, creatinine correction, metal excretion, mitochondria, photobiomodulation, urine toxic metals

## Abstract

**Background:**

Aluminum neurotoxicity has been implicated in neurodevelopmental disorders, including autism spectrum disorder (ASD). Nonchelation approaches that may influence urinary metal excretion remain largely unexplored. Photobiomodulation (PBM) at 660 nm modulates mitochondrial cytochrome c oxidase activity and cellular bioenergetics, with possible downstream effects on renal physiology.

**Case:**

A 5-year-old boy with ASD and a historical elevated urinary aluminum result (220 µg/g creatinine at age 3) underwent serial urine toxic metals testing.

**Methods:**

Eight spot urine specimens (November 7–19, 2025) were analyzed by ICP-MS/MS (Doctor's Data, Inc.) at ^27^Al, with creatinine by Jaffé reaction. Two baseline specimens preceded pulsed 660 nm PBM initiation. A follow-up specimen was collected January 21, 2026.

**Intervention:**

Pulsed 660 nm PBM via multi-diode red laser (Program #1: 9/33/60 Hz, 15 min/session, 3 × /day) on Days 3–8. Specimens collected as consistent first morning voids.

**Results:**

Baseline urinary aluminum was stable (10.0 and 9.8 µg/g creatinine; reference <40). After PBM, aluminum rose to 58 µg/g on Day 3 and peaked at 98 µg/g on Day 7. A severely dilute Day 8 specimen was excluded. At two-month follow-up, aluminum remained above reference (49 µg/g). Mercury exceeded reference in all specimens including baseline. Follow-up mercury rose to 8.4 µg/g creatinine with urine pH >8.0.

**Conclusion:**

This case describes a temporal increase in urinary aluminum excretion following pulsed 660 nm PBM. Urinary aluminum alone is insufficient to establish systemic mobilization, reduced tissue burden, or clinical benefit. The observation is hypothesis-generating; controlled studies with verified dosimetry and validated outcomes are warranted.

## Introduction

Aluminum is the most abundant metallic element in the Earth's crust and is ubiquitous in food, drinking water, consumer products, and certain vaccine adjuvants. Although not biologically essential, aluminum is a well-characterized neurotoxin. Chronic low-level exposure has been linked, in animal models, to oxidative stress, mitochondrial dysfunction, neuroinflammation, and disruption of glutamate and cholinergic neurotransmitter systems (1, 2).

Evidence of aluminum accumulation in the central nervous system of individuals with autism spectrum disorder (ASD) has emerged from postmortem brain studies. Mold et al. (2018) reported substantially elevated aluminum concentrations across multiple brain regions in individuals with ASD compared with age-matched neurotypical controls, with particularly high concentrations in inflammatory glial cells, suggesting ongoing neuroinflammatory processes (3). Complementary work by Adams et al. (2013) described differences in urinary metal excretion profiles between children with ASD and neurotypical peers (4). Together, these findings suggest that aluminum bioaccumulation may be relevant to the pathobiology of at least a subset of ASD cases.

Chelation therapy is the standard clinical approach for heavy metal mobilization, but carries meaningful risks in pediatric populations, including redistribution of metals to the central nervous system if applied without adequate monitoring, renal toxicity, and depletion of essential elements. Nonchelation strategies intended to influence metal mobilization and urinary excretion—through enhancement of endogenous metabolic and renal pathways—remain largely unexplored and are of potential clinical interest.

Photobiomodulation (PBM) is the application of low-level, non-thermal light in the red to near-infrared spectrum to modulate cellular function. The primary chromophore for red-spectrum PBM is cytochrome c oxidase (Complex IV of the mitochondrial respiratory chain). Absorption of 660 nm photons by cytochrome c oxidase dissociates inhibitory nitric oxide, increases electron transport chain activity, elevates mitochondrial membrane potential, and augments cellular ATP production (5, 6). Downstream consequences include shifts in redox balance, modulation of reactive oxygen species signaling, upregulation of glutathione synthesis, and altered gene expression profiles relevant to inflammation and cellular stress responses (5, 7).

PBM has been examined as a potential intervention in ASD, with recent mini-reviews and small clinical trials reporting behavioral and electrophysiological changes following transcranial PBM in pediatric and adult ASD populations (8–10). Preclinical work in valproic acid-induced rodent models of ASD has reported attenuation of cognitive dysfunction and neuroinflammation following 830 nm PBM (11). These studies have not, however, examined urinary metal excretion as an outcome.

Whether PBM influences metal handling is an open question. Plausible but unproven pathways include changes in the activity of ATP-dependent membrane efflux transporters of the ABC superfamily (e.g., MRP1/ABCC1, MRP2/ABCC2, P-glycoprotein/ABCB1) in the kidney and liver (12), alterations in glutathione-mediated metal conjugation, and PBM-induced shifts in renal perfusion or tubular handling. There is currently limited direct evidence that 660 nm PBM enhances heavy metal clearance in any system, mitochondrial activation does not necessarily imply increased metal transport or excretion, and no mechanistic biomarkers of these pathways are evaluated in the present case. The mechanistic discussion in this report is therefore framed as hypothesis-generating only.

Here, we report a pediatric case in which serial urinary aluminum increased following initiation of pulsed 660 nm PBM, with two pre-intervention baseline specimens and a two-month follow-up measurement. The primary aim of this report is to document the laboratory kinetics, describe the intervention parameters, and characterize the limitations relevant to future controlled investigation. We do not claim that the observed increase in urinary aluminum reflects reduced tissue or systemic burden, nor that it corresponds to any clinical benefit.

## Case presentation

The patient is a 5-year-old boy with a diagnosis of ASD, receiving care at Bogner Health LLC (Bloomfield Hills, MI, United States). Written informed consent was obtained from the boy's mother for de-identified case publication and further research use prior to any data collection for this report.

Relevant history includes a urinary aluminum value of 220 µg/g creatinine obtained at age 3 under the care of a separate clinician using an unspecified collection protocol and laboratory. This historical value is documented solely to establish a prior clinical concern for aluminum burden; it is not used for trend analysis or comparison with the present measurements, as differences in collection materials, timing, and analytical methodology preclude meaningful comparison.

### Aluminum exposure history

A structured aluminum exposure history was obtained from the boy's mother at enrollment. Maternal recall did not identify residence near aluminum smelting or processing facilities, regular use of aluminum-based cookware, antiperspirant use in the child, or known occupational exposure in either parent. Drinking water source during pregnancy, breastfeeding, and current household use was municipal supply; no private well water was used. Aluminum-containing antacids or buffered analgesics were not regularly administered. The boy received age-appropriate childhood immunizations on the standard schedule, several of which contain aluminum adjuvants per manufacturer labeling; specific brands and lot numbers were not retrievable at the time of this report. Maternal occupational and environmental exposure history during pregnancy was unremarkable per recall. We note that exposure history relies on maternal recall and was not independently verified through environmental sampling, dietary records, or aluminum content analysis of drinking water; this is acknowledged as a limitation.

### Behavioral and clinical observations during the protocol

Validated behavioral assessment instruments (e.g., CARS, ABC, ATEC), standardized adverse event reporting tools, and prospective diet and supplement diaries were not deployed for this case report; this is acknowledged as a key limitation and the report is framed accordingly as a laboratory kinetics observation rather than a treatment outcome study. Retrospective maternal observations during the 11-day protocol period did not identify acute adverse events, changes in feeding pattern, new gastrointestinal symptoms, sleep disturbance beyond baseline, or behavioral deterioration. No skin reaction, ocular complaint, or behavioral aversion to the device was observed. These observations are reported descriptively and should not be interpreted as evidence of clinical benefit or absence of harm; prospective capture using validated instruments is planned for the controlled study that this case report is intended to motivate.

## Methods

### Reporting guidance

This case report was prepared in accordance with CARE (CAse REport) guidelines, including explicit intervention timing, a laboratory trend figure, and direct statement of limitations.

### Urine collection and analysis

**Figure 1:**
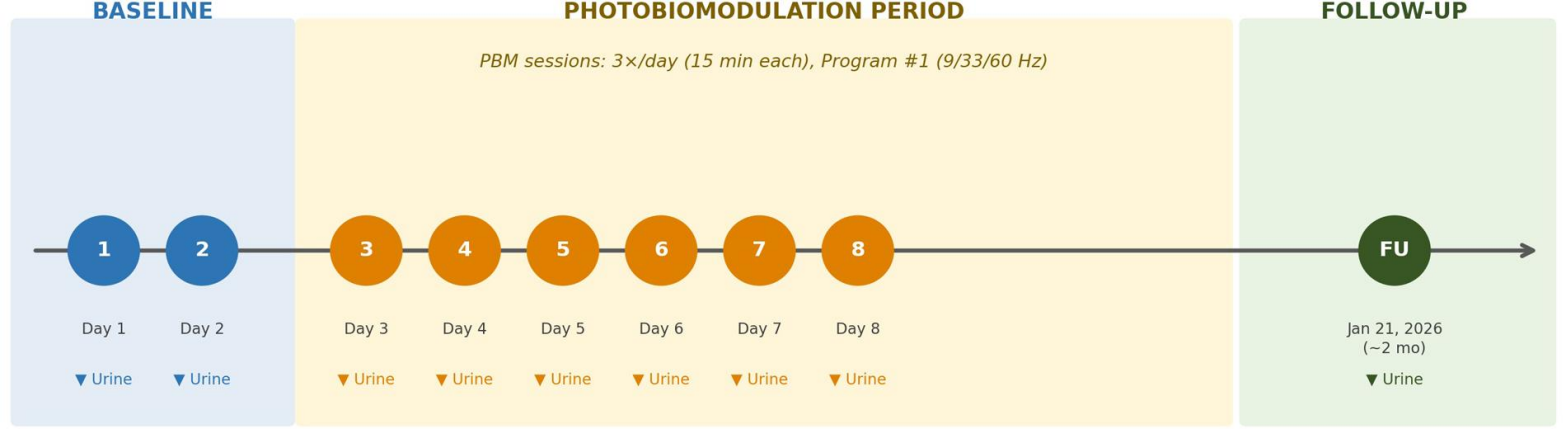
Protocol timeline. Baseline urine specimens collected on Days 1–2; pulsed 660 nm PBM administered on Days 3–8 (3 sessions/day, 15 min each, Program #1: 9/33/60 Hz); follow-up specimen collected January 21, 2026 (∼2 months post-protocol).

**Figure 2:**
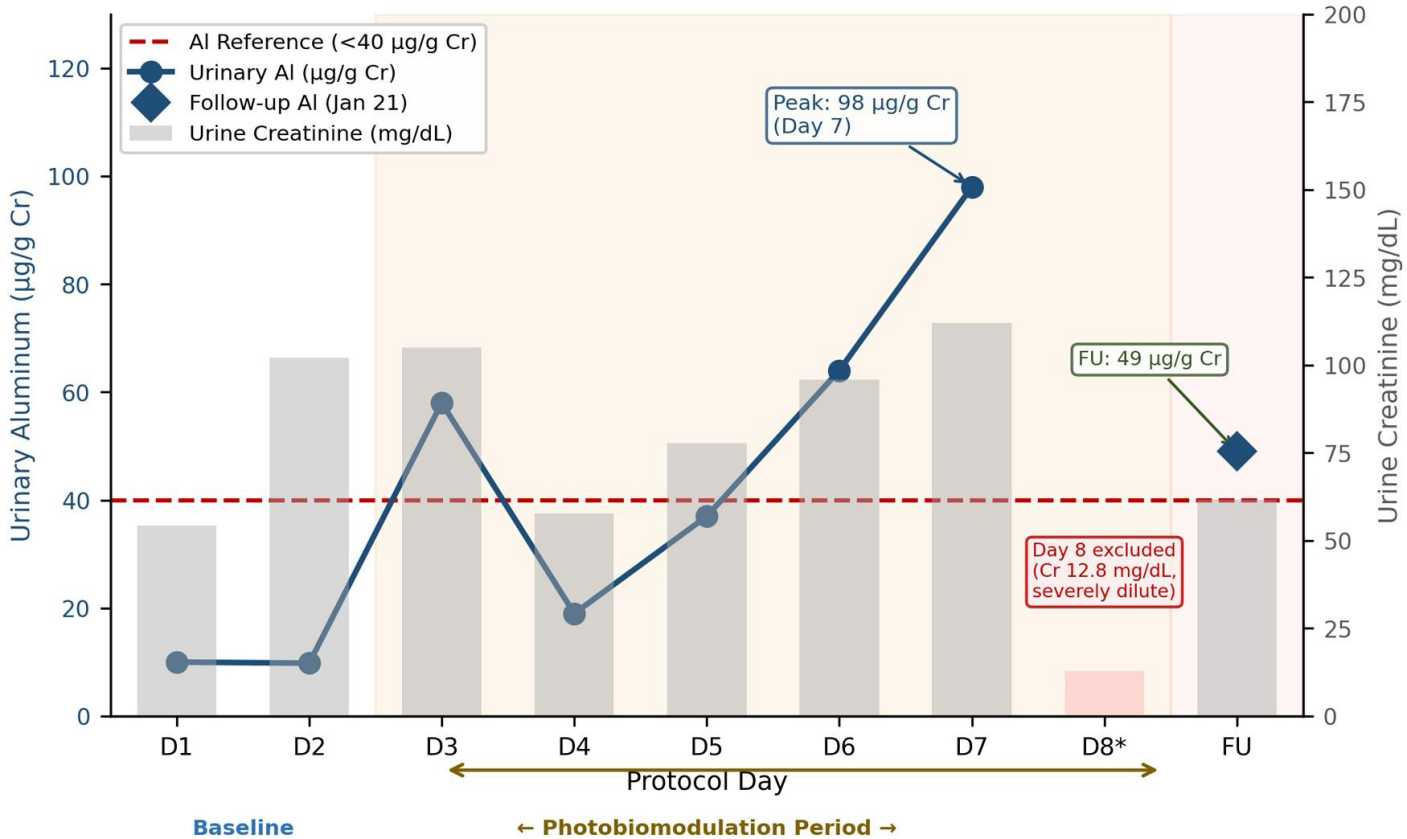
Urinary aluminum (µg/g creatinine, left axis, blue line with circles) and urine creatinine (mg/dL, right axis, gray bars) by protocol day. Red dashed line indicates laboratory reference interval for urinary aluminum (<40 µg/g creatinine). Day 8 pink bar indicates a severely dilute specimen (creatinine 12.8 mg/dL) excluded from primary interpretation. Diamond marker (◆) = follow-up specimen (January 21, 2026). PBM=photobiomodulation period (Days 3–8).

Eight spot urine specimens were collected during the protocol window (November 7–19, 2025) and sent to Doctor's Data, Inc. (St. Charles, IL, United States; CLIA ID: 14D0646470) for toxic metals analysis. Each specimen was collected at approximately the same morning time across all collection days, as the first void following the conclusion of the prior evening's third PBM session, providing a consistent diurnal collection interval and limiting circadian variability in urinary metal excretion. The protocol timeline is shown in Figure 1, and serial urinary aluminum and creatinine values are shown in Figure 2.

All specimens were collected in organic cotton-based kits intended to minimize aluminum contamination from collection materials; Doctor's Data, Inc. confirmed independent verification of low aluminum contamination for these kits (written confirmation on file with the corresponding author).

### Analytical method summary

Urine toxic metals were quantified by inductively coupled plasma tandem mass spectrometry (ICP-MS/MS), an analytical platform that uses two mass analyzers separated by a collision/reaction cell to remove polyatomic and isobaric interferences common in urine matrices. Aluminum was monitored at mass 27 (^27^Al), the only stable isotope of aluminum. Specimens were diluted and acidified per standard laboratory protocol, with internal standards added to control for matrix effects and instrument drift. Calibration was performed against NIST-traceable standards, with daily quality control specimens analyzed alongside patient samples. Mercury, arsenic, thallium, cesium, antimony, cadmium, tungsten, lead, and tin were quantified within the same analytical run. Urine creatinine was measured by the Jaffé reaction. Results are reported as µg/g creatinine.

### Specimen adequacy and creatinine correction

Creatinine correction was used to adjust for urine dilution. Per the laboratory reference range, specimens with creatinine below 25 mg/dL were considered severely dilute; creatinine-corrected values from those specimens were not used for primary interpretation. Day 8 creatinine was 12.8 mg/dL, and Day 8 creatinine-corrected values are therefore treated as non-interpretable for primary analysis. A dilution impact analysis via back-calculation and renormalization to creatinine 100 mg/dL is provided in Table 1 for transparency.

**Table 1:** Day 8 dilution impact analysis (renormalization to creatinine 100 mg/dL).

Metal	Reference (µg/g creatinine)	Reported D8 (creatinine 12.8 mg/dL)	Absolute (µg/L)	Renormalized to creatinine 100 mg/dL	Exceeds Reference?
Aluminum	<40	68	8.70	8.7	No
Cesium	<11	13	1.66	1.7	No
Antimony	<0.25	0.26	0.033	0.033	No
Cadmium	<0.16	0.16	0.020	0.020	No

Formula: Absolute (µg/L) = Reported (µg/g creatinine) × Creatinine (g/L). Creatinine 12.8 mg/dL = 0.128 g/L. Normalized=Absolute ÷ 1.0 (at creatinine 100 mg/dL = 1 g/L). No metal exceeds reference when corrected for dilution.

### Photobiomodulation intervention

Pulsed 660 nm PBM was administered using a multi-diode red laser device (DCL-6R; Researched Elements Tech LLC, Farmington Hills, MI, United States; six red laser diodes). Program #1 delivered dynamic frequency cycling at 9, 33, and 60 Hz at 2-second intervals. Each session was 15 min in duration. The protocol consisted of three sessions per day on protocol Days 3 through 8 (18 total sessions).

The choice of 660 nm wavelength reflects established absorption by cytochrome c oxidase as the primary red-spectrum photoacceptor (5, 6). The 9/33/60 Hz cycling regimen was selected based on the manufacturer's empirically derived program rather than from a formal frequency-response study; pulsed PBM has been proposed to offer biological advantages over continuous-wave delivery in selected applications (7, 13), but the specific 9/33/60 Hz combination has not been independently validated against alternative frequencies for this indication. This is acknowledged as a limitation and a target for systematic optimization in future controlled work.

Device output power, beam profile, spot size, irradiance, and fluence were not independently measured by calibrated instrumentation for the specific unit used in this case. Manufacturer-specified parameters for the DCL-6R (Researched Elements Tech LLC) are documented in Table 2; these represent nominal specifications. Operating distance and technique were applied per manufacturer guidelines (non-contact, approximately 1–2 cm from skin surface). Treated anatomic sites were not prospectively recorded in the clinical record. Independent dosimetry verification is required for replication and is itemized in Table 2.

**Table 2:** Photobiomodulation protocol parameters.

Parameter	This case	Notes for replication
Wavelength	660 nm	Red-spectrum PBM
Emission type	Pulsed laser diodes×6 (DCL-6R)	Class 2; <5 mW peak total
Pulse program	Program #1: 9/33/60 Hz; 2 s per step; 3:7 duty cycle (30% on)	Dynamic cycling; alternative pulsed and continuous-wave comparisons recommended
Session duration	15 min	—
Sessions per day	3	18 total sessions across Days 3–8
Protocol days	Days 3–8	Days 1–2 = urine baseline only
Output power	<5 mW peak (all 6 diodes combined); ∼<1.5 mW time-averaged at 30% duty cycle	Verify per-diode and total with calibrated power meter
Beam/spot size	6 line beams; 15° span; est. ∼0.1–0.3 cm^2^ illuminated area at 1–2 cm	Measure spot dimensions at treatment distance
Distance/technique	Non-contact; 1–2 cm (range 0–3 cm); per-session distance not logged	Record distance each session; specify contact vs. non-contact
Irradiance	Est. 5–15 mW/cm^2^	Measure at skin surface with calibrated detector
Fluence per session	Est. 4.5–13.5 J/cm^2^ (irradiance×900 s; not instrument-verified)	Report measured J/cm^2^ per site
Treated sites	Circumferential cranial: frontal, bilateral temporal/parietal, occipital; continuous motion; ≤60 s per point	Map and photograph sites each session with standardized anatomical notation
Safety/AE monitoring	Class 2; no protective eyewear required (IEC 60825-1:2014); beam avoidance maintained; no adverse events reported	Document AE monitoring criteria and observer role

Multiple dosimetry elements were not independently verified and are required for replication; these are noted in the third column.

The boy's mother reported no changes in diet, supplements, or new environmental aluminum exposures during the protocol period. This report is based solely on maternal recall and was not independently verified through dietary records, supplement logs, or environmental assessment.

## Results

Table 3 summarizes serial urinary metals and creatinine across all specimens. Baseline urinary aluminum was stable on Days 1 and 2 (10.0 and 9.8 µg/g creatinine, respectively), within the laboratory reference interval (<40 µg/g creatinine). After PBM initiation on Day 3, urinary aluminum increased to 58 µg/g creatinine, decreased on Days 4 and 5, then rose again on Days 6 and 7, peaking at 98 µg/g creatinine. Day 8 creatinine was 12.8 mg/dL; creatinine-corrected Day 8 values are excluded from primary interpretation (see Table 1 for dilution impact analysis). At two-month follow-up (January 21, 2026), urinary aluminum remained above reference at 49 µg/g creatinine.

**Table 3:** Serial urinary toxic metals. Element symbols used for table compactness; full element names are used throughout the manuscript text.

Metal (µg/g creatinine)	Ref	D1	D2	D3	D4	D5	D6	D7	D8*	FU
Aluminum (Al)	<40	10.0	9.8	58★	19	37	64★	98★	68★†	49★
Mercury (Hg)	<0.7	2.7★	1.8★	1.9★	3.0★	1.1★	1.2★	2.4★	2.2★†	8.4★‡
Arsenic (As)	<30	13	26	14	17	12	31★	27	15†	15
Thallium (Tl)	<0.5	0.53★	0.54★	0.63★	0.65★	0.43	0.35	0.37	0.29†	0.29
Cesium (Cs)	<11	8.2	10	8.8	7.5	9.9	11	9.1	13★†	8.6
Cadmium (Cd)	<0.16	0.03	0.03	0.04	0.07	0.04	0.06	0.06	0.16†	0.03
Antimony (Sb)	<0.25	0.10	0.11	0.10	0.23	0.075	0.10	0.077	0.26★†	0.079
Tungsten (W)	<0.6	0.22	0.18	0.076	0.077	0.72★	0.30	0.12	0.37†	0.11
Lead (Pb)	<1.1	0.55	0.45	0.36	0.51	0.50	0.58	0.43	0.73†	0.52
Tin (Sn)	<9	0.14	0.34	0.26	0.37	0.23	0.28	0.19	1.0†	9.5★‡
Creatinine (mg/dL)	25–180	54.3	102	105	57.7	77.8	95.9	112	12.8↓	61.7

★ = Exceeds laboratory reference interval (adequate creatinine specimens only).

† = Day 8 value from severely dilute specimen (creatinine 12.8 mg/dL; reference 25–180 mg/dL). Excluded from primary interpretation; see Table 2.

‡ = Follow-up specimen, urine pH >8.0. Alkaline urine may alter metal recovery; interpret with caution.

↓ = Creatinine below laboratory reference range for adequate specimen (< 25 mg/dL).

*The severely dilute Day 8 specimen (creatinine 12.8 mg/dL), excluded from primary interpretation.

Mercury exceeded reference in all specimens including baseline (Days 1–2: 2.7 and 1.8 µg/g creatinine; reference <0.7), confirming a pre-existing mercury burden independent of PBM initiation. Thallium was elevated above reference at baseline (Days 1–2: 0.53 and 0.54 µg/g creatinine; reference <0.5) and through Day 4, then normalized in later specimens—indicating that the thallium elevation predates the intervention. Single-day reference exceedances were observed for arsenic (Day 6) and tungsten (Day 5), consistent with dietary or environmental variability.

At follow-up, mercury rose markedly to 8.4 µg/g creatinine with concurrent urine pH >8.0. Tin was borderline elevated at 9.5 µg/g creatinine (reference <9) in the same specimen. These findings are discussed further below.

## Discussion

This case documents a temporal association between initiation of pulsed 660 nm PBM and an increase in urinary aluminum on days with adequate creatinine. The aluminum rise was evident on the first post-initiation specimen (Day 3; 58 µg/g creatinine) and peaked on Day 7 (98 µg/g creatinine), representing approximately a tenfold increase above stable baselines. Urinary aluminum remained above the laboratory reference interval at two-month follow-up. Because this is a single-subject observation without a sham control or randomization, the findings support a temporal association only and should be regarded as hypothesis-generating.

### Urinary excretion vs. systemic body burden

A central interpretive limitation of this case must be stated explicitly. Urinary aluminum concentration alone is insufficient to establish systemic aluminum mobilization, reduction in tissue burden, or evidence of “detoxification.” Urinary metal measurements can be affected by multiple confounders, including hydration status, dietary or environmental exposure, renal handling, urine pH, specimen collection timing, and analytical variability. An increase in urinary aluminum excretion is consistent with—but does not prove—mobilization from tissue stores; it may equally reflect altered renal handling of recently absorbed aluminum, transient redistribution between compartments without net change in total body burden, or analytical variation. Elevated urinary aluminum excretion also does not, by itself, indicate any reduction in neurological aluminum content or any clinically meaningful improvement. Distinguishing among urinary excretion measurements, systemic body burden, and clinically meaningful therapeutic benefit requires complementary biomarkers (e.g., serum or whole-blood aluminum, hair, provoked vs. unprovoked excretion comparisons), prospective validated behavioral assessment, and controlled study designs. None of these are available in the present case.

### Pattern of metal excretion across the protocol

Several metal excretion patterns argue against a non-specific global increase in urinary metals driven by a non-intervention confounder. Thallium elevation predated the intervention (elevated at baseline on Days 1–2) and normalized during the PBM period, suggesting an independent pre-existing burden unrelated to the PBM protocol. Arsenic and tungsten showed single-day elevations consistent with dietary or environmental variability rather than a sustained response. Day 8 values that appeared broadly elevated across multiple analytes were attributable to severe dilution effects, as confirmed by renormalization in Table 1, where no analyte exceeded its reference interval after correction.

### Mechanistic considerations

Possible but unproven mechanisms by which 660 nm PBM might influence urinary metal excretion include: (i) modulation of cytochrome c oxidase activity and downstream ATP availability, which could in principle affect ATP-dependent membrane efflux transporters of the ABC superfamily in renal and hepatic epithelia;⁵ (ii) altered glutathione dynamics affecting metal conjugation capacity and the bioavailability of metals for urinary excretion; (iii) PBM-induced shifts in renal perfusion, glomerular filtration, or tubular reabsorption, which could change net urinary clearance of filtered metals independently of any change in tissue burden; (iv) effects on systemic and tissue oxidative stress responses that may alter intracellular metal binding by metallothioneins and related ligands (14, 15); and (v) effects on lymphatic drainage and perivascular clearance that could mobilize metals from interstitial compartments without changing total body burden. We emphasize that no mechanistic biomarkers (e.g., transporter activity assays, glutathione levels, urinary metallothionein, renal hemodynamic measures) were evaluated in this case. Attribution of the observed aluminum increase to any single transporter or pathway is therefore speculative and unsupported by the data presented here.

### Follow-up mercury and tin

The follow-up specimen (January 21, 2026) showed a marked mercury increase to 8.4 µg/g creatinine—approximately three- to five-fold above the PBM-period values—accompanied by borderline-elevated tin at 9.5 µg/g creatinine (reference <9) and urine pH >8.0. This pattern warrants careful interpretation. Alkaline urine (pH >8.0) can affect the recovery and ionization of some metals during ICP-MS/MS analysis and may independently alter tubular metal handling. Importantly, the exposure history between the end of the protocol (Day 8, November 19, 2025) and follow-up (January 21, 2026) was not systematically characterized. Potential explanations for the mercury elevation include dietary seafood intake, changes in supplementation (particularly preparations containing methylcobalamin, lipoic acid, or glutathione, which are known to influence mercury mobilization), dental procedures, or new household exposures. The concurrent tin elevation is unexplained and may reflect a dietary source (e.g., canned foods) or an unidentified exposure. These findings underscore the importance of systematically documenting exposure history, supplementation changes, and dietary pattern at every measurement point in future studies. A repeat specimen with normal pH would be required to determine whether the mercury elevation represents a true persistent increase or a pH-related artifact. The treating clinician was informed of the follow-up mercury result and clinical reassessment was arranged; outcomes of that reassessment are outside the scope of this report.

### Clinical significance

The clinical significance of the laboratory observations reported here is, by design, limited. This is a laboratory kinetics report; validated behavioral assessment instruments (CARS, ABC, ATEC), standardized adverse event capture, sleep diaries, parent-rated gastrointestinal symptom scales, and cognitive testing were not deployed during the protocol. The report does not establish that the observed urinary aluminum increase corresponded to any change in autism severity, cognition, sleep, behavior, or core ASD symptoms. We have intentionally framed the report as motivating—not substituting for—a controlled study with prospective clinical outcome capture. Planned next-step work will include sham-controlled randomized assignment, validated ASD severity and behavioral instruments at multiple timepoints, prospective adverse event monitoring, dietary and supplement diaries, and independent dosimetry verification.

### Limitations

Key limitations include: (1) single-subject design with no sham PBM control and no randomization; (2) absence of prospective capture of clinical outcomes, adverse events, validated ASD severity metrics, sleep, diet pattern, and exposure history during the protocol; (3) absence of complementary biomarkers of systemic aluminum burden (serum or whole-blood aluminum, hair aluminum, provoked vs. unprovoked excretion comparisons); (4) incomplete dosimetry documentation, including the absence of independently measured output power, irradiance, fluence, treated sites, and contact technique; (5) reliance on maternal recall for aluminum exposure history without independent environmental verification; (6) absence of mechanistic biomarkers to distinguish among possible pathways linking PBM to urinary metal excretion; and (7) the manufacturer of the PBM device is affiliated with the corresponding authors, representing a potential conflict of interest, disclosed in full below. Future studies should independently verify device dosimetry; prospectively capture clinical trajectories using validated instruments; record medication, supplement, and dietary records; conduct systematic environmental exposure assessment; and incorporate complementary biomarkers of systemic metal burden.

## Conclusion

In a 5-year-old boy with ASD, pulsed 660 nm PBM temporally coincided with an approximately tenfold increase in urinary aluminum on days with adequate creatinine, with urinary aluminum remaining above the laboratory reference interval at two-month follow-up. We do not interpret this temporal association as evidence of systemic aluminum mobilization, reduced tissue burden, or clinical benefit. Concurrent mercury elevations at baseline and a marked unexplained mercury rise at follow-up highlight additional metal burden considerations in this patient. These observations are hypothesis-generating only. Controlled studies with independently verified dosimetry, prospective clinical outcome tracking using validated instruments, systematic exposure documentation, and complementary biomarkers of systemic metal burden are needed to evaluate causality and clinical relevance (16–18).

## Data Availability

The original contributions presented in the study are included in the article/Supplementary Material, further inquiries can be directed to the corresponding author.
